# Carotid artery stump pressure and associated neurological changes in predominantly symptomatic carotid artery disease patients undergoing awake carotid endarterectomy

**Published:** 2009-04

**Authors:** P Rajaruthnam, TV Mulaudzi, JV Robbs, N Paruk, B Pillay, BM Biccard

**Affiliations:** Durban Metropolitan Vascular Service, Durban; Durban Metropolitan Vascular Service and Department of Surgery, Nelson R Mandela School of Medicine, University of KwaZulu-Natal, Durban; Durban Metropolitan Vascular Service and Department of Surgery, Nelson R Mandela School of Medicine, University of KwaZulu-Natal, Durban; Durban Metropolitan Vascular Service and Department of Surgery, Nelson R Mandela School of Medicine, University of KwaZulu-Natal, Durban; Durban Metropolitan Vascular Service and Department of Surgery, Nelson R Mandela School of Medicine, University of KwaZulu-Natal, Durban; Department of Anaesthesiology, Nelson R Mandela School of Medicine, University of KwaZulu-Natal, Durban

## Abstract

**Aim:**

To determine the mean carotid artery stump pressure (SP) at which patients develop neurological changes while undergoing awake carotid artery endarterectomy (CEA) under cervical block anaesthesia (CBA).

**Methods:**

A prospective analysis was carried out of patients undergoing awake CEA under CBA between February 2004 and April 2007. All patients had mean SP measured, with selective shunting on those who developed neurological symptoms on carotid artery clamping regardless of stump pressure. A ball connected to a pressure sensor was put in the patient’s contra-lateral hand.

**Results:**

Fifty-nine patients had awake CEA, 40 were males with a mean age of 64 years. Indications for CEA were asymptomatic high-grade stenosis in 12 (20%) patients and symptomatic stenosis in 47 (80%). Seven (12%) patients required shunting, one for transient ischaemic attack (TIA) and six for loss of consciousness. Six of these patients had presented with symptomatic disease.

Taking the threshold of mean carotid SP of 50 mmHg as an indication for shunting, 22% (6/27) of patients with a mean SP of < 50 mmHg required shunting and only 3% (1/32) with a mean carotid SP of > 50 mmHg needed a shunt. This was not statistically significant. Using a mean carotid SP of ≤ 40 mmHg as the threshold for shunting, 40% (4/10) of patients required shunting and 3% (1/31) with a mean carotid SP of > 40 mmHg required shunting. This was statistically significant. Thirteen (22%) patients were complicated by transient hoarseness of voice. One (2%) had a haematoma that required re-exploration. None of these patients had any major postoperative neurological or cardiological complications.

**Conclusion:**

Even though the sample in this study was small, awake CEA under local anaesthesia was seen as a safe procedure. It would appear to be safe to use the mean SP of 40 mmHg as a threshold for selective shunting in CEA under general anaesthesia.

## Summary

Awake carotid endarterectomy (CEA) performed under cervical block anaesthesia (CBA) is now at least as safe and effective as that performed under general anesthesia. It is possible that awake CEA results in less shunting, fewer neurological complications, decreased surgical time,^1^-^3^ fewer admissions to intensive care and shorter hospital stays,^3^-^5^ although there is currently insufficient prospective data to confirm this.^6^ CEA under CBA may also result in haemodynamically more stable patients.^2^,^3^,^7^ Interestingly, CEA under CBA has been shown to be associated with less peri-operative myocardial ischaemia^8^,^9^ and possibly fewer cardiopulmonary complications.^2^,^3^,^9^,^10^ Awake CEA may also allow the patient to alert the anaesthetist to chest pain.^11^

Although studies suggest that the best determinant of the need for shunt placement during CEA is an awake patient under CBA,^12^ some patients may still require CEA under general anaesthesia for various reasons. These may include anxiety, neck fixation, a short neck with a high carotid bifurcation, redo CEA and claustrophobia.^13^

Controversy still exists on whether to shunt all patients, or to shunt selectively when performing CEA under general anaesthesia. For those who prefer selective shunting, there is still no agreement on the best method to use for patient selection. Most surgeons use electroencephalography (EEG) and/or mean stump pressure to assess the adequacy of collateral cerebral blood flow.^12^-^19^ A mean SP < 50 mmHg has been suggested as a marker of the necessity to shunt.^16^,^19^ However, a recent analysis of awake CEA suggests that in patients whose presentation is asymptomatic, an SP < 40 mmHg may be a cost-effective alternative for selective shunting under general anaesthesia.^13^ A second study confirmed this finding.^12^ However, as 74 to 77% of the patients in these studies had asymptomatic carotid artery disease,^12^,^13^ it was suggested that an SP < 40 mmHg could not be recommended for symptomatic carotid artery disease until further research was conducted.^13^

This was a prospective review of our patients who had an awake CEA under CBA, to assess the SP at which patients develop neurological symptoms requiring shunting, particularly in patients presenting with symptomatic carotid artery disease.

## Patients and methods

Ethical approval was obtained to analyse patients undergoing awake CEA under CBA between February 2004 and February 2008 at Inkosi Albert Luthuli Central Hospital, Durban, South Africa. A total of 395 occlusive carotid disease interventions were performed during this period. One hundred of these interventions were carotid artery stenting (CAS), 239 were CEA performed under general anaesthesia and 59 were CEA under CBA. The 59 patients who had their CEA under CBA were analysed. The following data were documented: demographic data, indications for CEA, the degree of contra-lateral carotid artery disease, the mean SP, the presence of neurological symptoms on carotid clamping, insertion of shunts, and outcome.

All the patients were extensively counselled and consent was obtained for surgery under local anaesthesia. Most of the carotid endarterectomies in our institution are performed under general anaesthesia, and local anaesthesia is used based on the surgeon’s preference. Patients excluded were those with neck fixation, a short neck with a high carotid bifurcation, redo CEA, anxiety and claustrophobia.

All patients had a radial arterial line inserted for systemic blood pressure monitoring. Local anaesthesia included both superficial and deep cervical blocks with intra-operative supplementation of 2% lignocaine when necessary. A ball was placed in the patient’s contra-lateral hand and was transduced with a pressure manometer in order to generate a waveform on the anaesthetic monitor following compression of the ball.

Systemic heparin was given before carotid artery clamping. A 19-gauge needle was used to measure the SP. The systemic common carotid artery and radial arterial pressures were correlated before common carotid artery clamping. Both common and external carotid arteries were clamped to measure SP and to assess any ischaemic neurological deficit. This was done by constantly talking to the patient and asking him/her to squeeze the ball in the contra-lateral hand.

It is our practice to keep the mean systemic blood pressure approximately 10 mmHg above the patient’s baseline blood pressure, to ensure an adequate perfusion pressure during carotid cross clamping. Should the patient develop neurological symptoms on cross clamping, the systemic blood pressure is further augmented with vasopressors. If this does not reverse the neurological symptoms, a shunt is inserted. Postoperatively, all patients were managed in ICU for at least 24 hours.

Statistical analysis was conducted using a two-tailed Fisher’s exact test using Epicalc 2000 Version 1.02 (Gilman J, Myatt M, Brixton Books, 1998).

## Results

Fifty-nine patients were included in this review, 40 of whom were males. Their ages ranged from 46 to 82 years with a mean age of 64 years. Indications for surgery were asymptomatic highgrade stenosis in 12 (20%) patients and symptomatic stenosis in 47 (80%). The symptoms were transient ischaemic attack (TIA) in 23 (39%) and stroke in 24 (41%) of the 59 patients.

Seven (12%) patients required shunting for neurological deficit on carotid artery clamping, one for TIA and six for loss of consciousness (Table 1). Six patients who required shunting had presented with symptomatic disease, five had had a stroke and one a TIA. One patient with asymptomatic disease required shunting. Therefore, shunting was indicated in 13% (6/47) of the patients who had symptomatic disease.

**Table 1:** Indications For Shunting

*Patients (n = 7)*	*Presenting symptoms*	*Contra-lateral ICA*	*Mean SP (mmHg)*	*Indications for shunting*
Male	TIA	Occluded	35	TIA
Male	Stroke	Normal	40	LOC
Female	Stroke	45% stenosis	31	LOC
Female	Stroke	60% stenosis	68	LOC
Female	Stroke	90% stenosis	34	LOC
Female	Stroke	60% stenosis	30	LOC
Female	Asymptomatic	50% stenosis	18	LOC

ICA: internal carotid artery, SP: stump pressure, TIA: transient ischaemic attack, LOC: loss of consciousness.

Taking the threshold of the mean carotid SP of 50 mmHg as an indication for shunting (Table 2), 22% (6/27) of patients with a mean carotid SP of < 50 mmHg required shunting. Only 3% (1/32) of patients with a mean carotid SP of > 50 mmHg needed a shunt. This patient developed loss of consciousness on carotid artery clamping despite the mean carotid SP of 68 mmHg and a pulsatile waveform. This was not statistically significant with the Fisher’s exact test, with a *p*-value of 0.063.

**Table 2:** Need For Shunting Based On Mean Stump Pressure ≤ 50 and ≤ 40 MMHG

	*shunt n = 7*	*No shunt n = 52*	*Total n = 59*
SP ≤ 50 mmHg	6	21	27
SP > 50 mmHg	1	31	32
SP ≤ 40 mmHg	6	8	14
SP > 40 mmHg	1	44	45

Fisher’s exact test for SP ≤ 50 mmHg; *p* = 0.063 and for SP ≤ 40 mmHg; *p* = 0.0004.

Had carotid endarterectomy been done under general anaesthesia, the shunt rate would have been 41% (17/41) using the mean carotid SP of < 50 mmHg as a threshold. However, the one patient who required a shunt with a mean SP of 68 mmHg would have been missed.

Using a mean carotid SP of ≤ 40 mmHg as a threshold for shunting (Table 2), 40% (4/10) of patients required shunting, which was almost double the patients when using a mean SP < 50 mmHg as a threshold. Three per cent (1/31) of those with a mean carotid SP of > 40 mmHg required shunting. This was statistically significant with the Fisher exact test, with a *p*-value of 0.009.

Had CEA been done under general anaesthesia with a mean SP ≤ 40 mmHg as a threshold for shunting, the shunt rate would have been 24% (14/59) compared with the 46% (27/59) when a mean SP of 50 mmHg was used. An SP ≤ 40 mmHg had a sensitivity of 86% and a specificity of 85%.

Thirteen (22%) patients were complicated by transient hoarseness of voice. This was probably due to the deep cervical block. One patient (1.69%) had a haematoma that required re-exploration and this was done under general anaesthesia. None of these patients had any major postoperative neurological complications. There were no postoperative myocardial infarctions or deaths.

## Discussion

The SPs obtained during awake CEA have been used to propose therapeutic indications for shunting under general anaesthesia.^11^ A mean SP < 40 mmHg has been shown to be a more appropriate pressure for shunt insertion in patients with asymptomatic carotid artery disease.^10^,^11^ It is unknown whether a mean SP < 40 mmHg is an appropriate indication for shunt insertion in patients with predominantly symptomatic carotid artery disease. Our study suggests that a mean SP ≤ 40 mmHg might be an appropriate indication for shunt insertion under general anaesthesia, provided that the mean systemic blood pressure is also maintained during carotid cross clamping.

Twelve per cent of the patients developed neurological symptoms during carotid cross clamping, which was similar to that in other studies.^5^,^10^,^11^ However, analysis of the symptomatic patients alone suggests that patients with symptomatic carotid artery disease may be more likely to require shunt insertion.^10^,^11^ Our own experience with a symptomatic patient who underwent CEA under general anaesthesia showed a shunt rate of 36%.^18^ This was triple the shunt rate of CEA under CBA.

Our study suggests that use of a mean SP < 50 mmHg may not be sensitive or specific enough for selective shunting in symptomatic carotid artery disease when CEA is performed under general anaesthesia.

The major limitation of this study was the small sample size. However, statistically and clinically, the results presented here do not suggest that it would be appropriate to use a mean SP < 50 mmHg instead of ≤ 40 mmHg as an indication for shunt insertion. The mean SP of ≤ 40 mmHg could also be used as threshold for shunting in symptomatic patients. Compared with our previous experience, awake CEA has a lower shunt rate, it is a safe procedure and it appears to have lower cardiovascular and neurological complications.
